# Different approaches for dealing with rare variants in family-based genetic studies: application of a Genetic Analysis Workshop 17 problem

**DOI:** 10.1186/1753-6561-5-S9-S78

**Published:** 2011-11-29

**Authors:** Marcio Augusto Alfonso de Almeida, Andrea Roseli Vançan Russo Horimoto, Paulo Sérgio Lopes de Oliveira, José Eduardo Krieger, Alexandre da Costa Pereira

**Affiliations:** 1 Laboratory of Genetic and Molecular Cardiology, Heart Institute, University of Sao Paulo Medical School, Av. Dr. Eneas C Aguiar, 44-10 andar, São Paulo 05403-000, Brazil; 2 National Laboratory of Biosciences, Campinas, Caixa Postal 6192, São Paulo, CEP 13083-970, Brazil

## Abstract

Rare variants are becoming the new candidates in the search for genetic variants that predispose individuals to a phenotype of interest. Their low prevalence in a population requires the development of dedicated detection and analytical methods. A family-based approach could greatly enhance their detection and interpretation because rare variants are nearly family specific. In this report, we test several distinct approaches for analyzing the information provided by rare and common variants and how they can be effectively used to pinpoint putative candidate genes for follow-up studies. The analyses were performed on the mini-exome data set provided by Genetic Analysis Workshop 17. Eight approaches were tested, four using the trait’s heritability estimates and four using QTDT models. These methods had their sensitivity, specificity, and positive and negative predictive values compared in light of the simulation parameters. Our results highlight important limitations of current methods to deal with rare and common variants, all methods presented a reduced specificity and, consequently, prone to false positive associations. Methods analyzing common variants information showed an enhanced sensibility when compared to rare variants methods. Furthermore, our limited knowledge of the use of biological databases for gene annotations, possibly for use as covariates in regression models, imposes a barrier to further research.

## Background

The aim of genome-wide association studies (GWAS) is to determine genetic patterns that underlie human traits of medical interest. Despite their relative, the combined effect of the identified genetic variants accounts for only a small portion of the heritability for complex traits, such as hypertension or cancer [1]. Rare variant single-nucleotide polymorphisms (SNPs) are becoming the current candidates for explaining this missing heritability paradox. Because of their low frequency, some variants are almost family specific; their detection requires sufficiently large samples, dedicated platforms, and new statistical methods [2]. Deep-sequencing platforms are sensitive to detecting these variants, and the widespread use of these platforms will result in an immense number of rare variants detect [3].

Several approaches have been recently proposed to analyze rare genetic variants information. A common way to model is to count their relative abundance in case and control groups [4]. Hypothetically, alleles showing a higher frequency in case subjects are more likely to harbor at least one causal variant. Arguably, several more sophisticated approaches ought to be considered. Methods for common variants analysis (minor allele frequency [MAF] > 0.01) are well established, and their utility for rare variants needs to be evaluated. Using the Genetic Analysis Workshop 17 (GAW17) family data set, we explored and compared different approaches to account for both common and rare variants for traits of interest in the GAW17 phenotype simulation [5].

## Methods

### GAW17 data set

We classified each variant on the basis of its MAF supplied by the GAW17 snp_info file; a SNP was classified as a rare variant (MAF < 0.01;) otherwise as a common variant. We carried out all analyses using the family-based data set and the first family-based phenotype simulation (fam_phen.1). The GAW17 data set consists of 24,487 genetic variants (6,356 common and 18,131 rare variants) located in 3,204 genes. We used the gene_info file to annotate genes harboring putative associated variants. The sample is composed of 697 individuals distributed in eight extended pedigrees, and we performed the analyses using the binary variable, affected versus unaffected.

### Heritability estimates

We calculated polygenic heritability estimates for the affection status and quantitative traits Q1, Q2, and Q4 with Sex, Age, and Smoking status as covariates in each model. Using the variance components approach implemented in the SOLAR package [6], we calculated the heritability as the total phenotypic variance proportion explained by additive genetic effects after accounting for covariates. We scaled measures of qualitative traits (e.g., affection and Smoking status) so that the regression coefficient represents the effect of having the covariate present as opposed to absent.

### Family-based association analysis

The GAW17 allele files were formatted for the QTDT software [7]. The identity-by-descent (IBD) values were made available by the GAW17 data providers. The pairwise IBD matrices were gene specific and were encoded as 0, 0.5, or 1 (denoting sharing of 0, 1, or both alleles identical by descent). Using direct association and variance component analysis, we carried out four different QTDT analyses combining the information provided by common or rare variants.

### Identifying candidate genes using polygenic additive models

We obtained heritability estimates for affection status using Age, Q4, and the information provided by rare and common variants in each analyzed gene. We tested four different ways to deal with such information. In the first analysis, the total sum of rare alleles (synonymous and nonsynonymous) was used as a continuous covariate in the polygenic model. In the second analysis, only nonsynonymous rare variants were considered; individuals were coded as 0 for those homozygous for the wild-type allele of a particular variant or as 1 for those presenting at least one rare allele. The third analysis was based on counting the absolute number of minor alleles in each common SNP from a gene and using this variable as a covariate in the polygenic model. In the fourth analysis, the pedigrees were considered separately and each SNP was used as a covariate in a polygenic model. Variants reducing trait heritability in at least one family were selected.

### VEGAS analysis

VEGAS (versatile gene-based association study) suite combines the HapMap Phase 2 haplotypic information with association *p.values* of markers to establish a gene-based *p*–value [8]. Initially, we defined the set of GAW17 SNPs that could be mapped to the HapMap phase 2 YRI data set, which is composed of 8,850 variants.

### Linkage analysis

We performed linkage analyses using the SOLAR package [6]. To compose the map file, we used Haldane functions to map chromosomal locations (in centimorgans). Then we conducted two-point and multipoint linkage analyses for each of the 22 chromosomes. The linkage analysis used an interval of 5 cM and a fine-map parameter of 0.5.

### KEGG pathway analysis

A reliable analytical approach can disentangle a complex phenotype by presuming that associated genes share functional characteristics and probably belong to the same biological pathways. The Kyoto Encyclopedia of Genes and Genomes (KEGG) database organizes biological pathways knowledge. Initially, we downloaded the latest database release (September 1, 2010) and determined which genes were annotated in the KEGG database. We found that 1,032 genes were mapped in 189 pathways (a gene could participate in more than one pathway). Annotated genes represent only a third of the genes present in the GAW17 data set. A hypergeometric test were use to determined overrepresented pathways [9].

### Computer environment

All analyses were carried out in a computational environment composed of 24 Intel i7 computers summing a total of 192 computing nodes. This cluster is managed by a Linux Rocks distribution specially.

## Results

### Characteristics of the phenotype and polygenic analysis

We focused on trying to understand the genetic determinants of the main phenotype, affection status, which has a prevalence of approximately 30%. Using unrelated individuals from the first case-control data set, we were able to observe that affection was significantly associated with Age (odds ratio [OR], 1.07 per year; range, 1.05–1.08), Q1 (OR, 7.3; range 5.34–9.96), Q2 (OR, 2.6; range, 2.16–3.23), Q4 (OR, 0.2; range, 0.19–0.30), and current Smoking (OR, 2.0; range, 1.41–2.28). In a multivariate logistic regression model (using only independent samples), we tested the association of these variables and Affection Trait. . The results as follows: Age (*p* = 0.06, OR 1.04), Smoking status (*p* = 0.04, OR 2.25), Q1 (*p* < 0.001, OR 20.1), Q2 (*p* < 0.001, OR 20.6), and Q4 (*p* < 0.001, OR 0.06). Trait heritability varied depending on the model specified; without any covariates the heritability was only 0.10 (SE 0.07), but we observed higher values when adjusting for different covariates, especially for Age and Q4 (0.58) (Table 1). We used this last model with Age and Q4 as covariates in future mapping and association efforts because it maximized the trait’s heritability compared with a model without covariates and because it was more parameterized.

**Table 1:** Heritability estimates for the affection status from polygenic models with different covariates

Model	*h*^2^ (SE)	SC	KL
No covariance	0.10 (0.07)	–	–
Age, Sex, Smoking, and interactions	0.55 (0.16)	Age, Smoking	0.27
Q1, Q2, Q4, and interactions	0.06 (0.18) (ns)	Q1, Q2, Q4	0.63
Age	0.53 (0.15)	Age	0.24
Smoking	0.11 (0.08)	Smoking	0.02
Q1	0.17 (0.13) (ns)	Q1	0.32
Q2	0.005 (0.07) (ns)	Q2	0.12
Q4	0.60 (0.15)	Q4	0.27
Q1 × Q2	0.03 (0.07) (ns)	Q1 × Q2	0.03
Q1 × Q4	0.15 (0.08)	Q1 × Q4	0.02
Q2 × Q4	0.11 (0.06)	Q2 × Q4	0.01
Q4, Age	0.58 (0.15)	Q4, Age	0.27
Q1, Q2	0.13 (0.13) (ns)	Q1, Q2	0.39
Q2, Age	0.42 (0.17)	Q2, Age	0.44

The *h*^2^ column contains the heritability estimates of the affection status using the model defined in the model column. The SC column lists the covariates that were found to be significant in the model. The KL column gives the Kullback-Leibler *R*^2^ statistic. ns, nonsignificant.

### Linkage analysis

Modest signals of linkage were observed in chromosomes 3, 5, and 11 with LOD scores of 1.03, 1.29, and 1.52, respectively. Any one of these results would be acceptable for a follow-up study. These somewhat modest results could be due to the limited number of individuals in the cohort and the use of a binary trait reducing statistical power of this approach.

### Associated genetic variants through polygenic models

A set of 15 causal genes was used to simulate individual phenotypes. and we determined the sensitivity, specificity, positive predictive value, and negative predictive value of each strategy based on this set [10] (Table 2). The most significant *p*-value for each causal gene observed in each analysis is shown in Table 3,. The first analysis (Table 2, column A) used the absolute count of rare variants as a covariate, and it presented low sensitivity and low positive predictive Only one true causal gene (*PTK2*) was detected (Table 3, column A). Only 19 of the detected genes could be annotated in 16 KEGG pathways (8 of them enriched).

**Table 2:** Sensitivity, specificity, and positive and negative predictive values of each tested approach

	A	B	C	D	E	F	G	H
Sensitivity	0.066	0.066	0.133	0.600	0.066	0.133	0.133	0.133
Specificity	0.987	0.984	0.978	0.761	0.994	0.993	0.979	0.978
Positive predictive value	0.020	0.025	0.029	0.001	0.052	0.095	0.029	0.028
Negative predictive value	0.995	0.995	0.995	0.997	0.995	0.995	0.995	0.995

Each column represents a tested approach: (A) Using the absolute sum of rare variants as a covariate in the polygenic model, using the entire cohort. Selected genes were those that significantly reduced the trait heritability. (B) Using the absolute sum of nonsynonymous rare variants as a covariate in the polygenic model, using the entire cohort. Selected genes were those that significantly reduced the trait heritability. (C) Counting the absolute number of minor alleles in each common variant from a gene and using the gene as a covariate in the polygenic model. (D) Using the number of minor alleles present in common variants as a covariate in the polygenic model, using each family separately. Selected variants were those that significantly reduced the trait heritability in at least one family. (E) QTDT results using the common variants and a linear model of association. (F) QTDT results using common variants and the variance components model. (G) QTDT results using rare variants and a linear model of association. (H) QTDT results using rare variants and the variance components model.

**Table 3:** Observed *p*-values of causal genes in each tested approach and, when applicable, the genetic variant detected

Gene	A	B	C	D	E	F	G	H
*AKT3*	0.52, NA −0.74 (0.16)	0.52, NA −0.74 (0.16)	NCV	NCV	NCV	NCV	1, NA NC (0.16)	1, NA NC (0.16)
*BCL2L11*	0.53, NA −0.21 (0.22)	0.96, NA −0.02 (0.22)	0.74, NA −0.03 (0.27)	0.43, C2S2309 0.17 (0.27)	1, NA NC (0.27)	1, NA NC (0.27)	1, NA NC (0.27)	1, NA NC (0.27)
*ELAVL4*	0.01*, NA −0.76 (0.21)	0.98, NA −0.02 (0.21)	0.28, NA −0.12 (0.30)	0.01, C1S3201 0.46 (0.30)	1, NA NC (0.30)	1, NA NC (0.30)	1, NA NC (0.30)	1, NA NC (0.30)
*HSP90AA1*	0.09, NA −0.30 (0.04)	0.32, NA −0.41 (0.04)	0.63, NA −0.06 (0.04)	0.008, C14S3706 0.20 (0.04)	0.29, C14S3706 −0.03 (0.04)	0.21, C14S3706 −0.15 (0.04)	1, NA NC (0.04)	1, NA 0.14 (0.04)
*NRAS*	1, NA 0.00 (0.18)	1, NA 0.00 (0.18)	NCV	NCV	NCV	NCV	1, NA NC (0.18)	1, NA NC (0.18)
*PIK3C2B*	0.61, NA 0.05 (0.18)	0.69, NA −0.06 (0.18)	0.45, NA 0.12 (0.30)	0.0006, C1S9210 1.47 (0.30)	1, NA NC (0.30)	1, NA NC (0.30)	0.01, C1S9183 0.13 (0.30)	0.04, C1S9183 0.11 (0.30)
*PIK3C3*	1, NA 0.00 (0.28)	1, NA 0.00 (0.28)	0.23, NA −0.63 (0.34)	0.27, C18S2479 0.02 (0.34)	1, NA NC (0.34)	1, NA NC (0.34)	1, NA NC (0.34)	1, NA NC (0.34)
*PIK3R3*	0.12, NA 5.16 (0.17)	1, NA 0.00 (0.17)	0.23, NA −0.11 (0.17)	0.08, C1S2903 0.30 (0.17)	0.005, C1S2903 0.02 (0.17)	0.005, C1S2903 0.02 (0.17)	0.003, C1S2864 0.15 (0.17)	0.003, C1S2864 0.15 (0.17)
*PRKCA*	0.82, NA −0.21 (0.10)	1, NA 0.00 (0.10)	0.24, NA 0.11 (0.17)	0.035, C17S3578 0.64 (0.17)	0.15, C17S4567 0.04 (0.17)	0.15, C17S4567 0.04 (0.17)	1, NA NC (0.17)	1, NA NC (0.17)
*PRKCB1*	0.47, NA 0.15 (0.23)	0.62, NA 0.27 (0.23)	0.75, NA 0.02 (0.23)	0.11, C16S1808 0.29 (0.23)	1, NA NC (0.23)	1, NA NC (0.23)	1, NA NC (0.23)	1, NA NC (0.23)
*PTK2*	0.04, NA −1.62 (0.03)	0.04, NA 0.27 (0.03)	0.17, NA −0.28 (0.03)	0.029, C8S4830 0.26 (0.03)	1, NA NC (0.03)	1, NA NC (0.03)	1, NA NC (0.03)	1, NA NC (0.03)
*PTK2B*	0.89, NA 0.08 (0.13)	1, NA 0.00 (0.13)	0.003, NA −0.22 (0.20)	0.01, C8S911 −0.14 (0.20)	0.001, C8S911 0.05 (0.20)	0.001, C8S911 0.05 (0.20)	1, NA NC (0.20)	1, NA NC (0.20)
*RRAS*	0.41, NA −0.19 (0.17)	0.61, NA −0.42 (0.17)	0.33, NA 0.10 (0.20)	0.08, C19S4940 0.07 (0.20)	1, NA NC (0.20)	1, NA NC (0.20)	1, NA NC (0.20)	1, NA NC (0.20)
*SHC1*	0.71, NA −0.35 (0.09)	1, NA 0.00 (0.09)	0.15, NA −0.17 (0.09)	0.03, C1S7055 0.00 (0.09)	1, NA NC (0.09)	1, NA NC (0.09)	1, NA NC (0.09)	1, NA NC (0.09)
*SOS2*	1, NA 0.00 (0.25)	1, NA 0.00 (0.25)	0.008, NA −0.26 (0.28)	0.009, C14136 −0.21 (0.28)	1, NA NC (0.28)	1, NA NC (0.28)	1, NA NC (0.28)	1, NA NC (0.28)

Each cell gives four pieces of information: In the top line, the *p*-value and the associated genetic variant resulting from a nonconvergent model (or NA if it was not applicable) are given. An asterisk next to the *p*-value indicates that the gene was significant but enhanced the trait’s heritability. In the second line of each cell, the regression *β* value obtained in each analysis and the *β* value used by the GAW17 organization in the data simulation (in parentheses) are given; NC indicates genes not presenting a convergent QTDT model. NCV (no common variants) in a cell indicates that the gene did not present any common variants. Columns A and B were compared to the mean value of *β* of the simulated SNPs, and columns C–H were compared to the top *β* among simulated SNPs. The tested approaches A–H are defined in Table 2.

In the second analysis, we created a binary variable indicating whether or not a transcript carried a nonsynonymous variant. It was expected that the addition of biological information would enhance the specificity of the polygenic model (Table 2, column B). Thirty-nine genes were detected, but only one causal gene, *PTK2*, and six KEGG pathways were considered enriched. Nonsynonymous variants selection is rather simplistic because it ignores a large proportion of synonymous variants that may be important for gene regulation. The *p*-values of the causal genes obtained in this analysis also show that the biological information did not assist in the detection of causal genes (Table 3). Indeed, with the exception of *PTK2*, all *p*-values were equal to or greater than those obtained with the first approach (Table 3, columns A and B). Rare genetic variants have a low population frequency and sometimes are family specific. To detect such variants, we proposed an alternative approach that relied on separating pedigrees and using the polygenic model to detect rare variants that altered trait heritability in at least one family. This analysis was not successful because many variance component models did not converge. This could be credited to an analytical limitation imposed by a binary trait and the limited number of individuals possessing rare variants (data not shown).

Using the same strategy, we investigated the information provided by common variants by counting the number of minor alleles in each gene for each individual in the cohort. Sixty-seven genes were found to be associated, and only two of them were causal genes (*PTK2B* and *SOS2*), that were not detect by any rare variant approach (Table 3, column C). Three KEGG pathways could be considered enriched. In the last polygenic model tested, we searched for SNPs carrying allelic information that significantly reduced trait heritability in at least one family. This approach had significantly higher sensitivity (60%) compared to the previous approaches, but it was compromised by low positive predictive value because 767 genes were detected (Table 2, column D).

### QTDT association analysis

We used QTDT to test for association using a linear model of association and the parental information as covariates in separate sets of common and rare variants. When we inspected the common variants panel, we detected only one causal gene (*PTK2B*). This first tested QTDT approach presented low sensitivity but a significant increase in the positive predictive value (0.05) because only 19 genes showed evidence of association (*p* < 0.05) (Table 2, column E). We next applied the VEGAS approach and found that 135 genes with only 2 causal genes (*PIK3R3* and *PTK2B*) were considered significant. The same panel was inspected using a variance components approach with the same regression model applied to the polygenic model (Q4 and Age as covariates). Twenty-one genes and two causal genes were detected. This approach had similar sensitivity and specificity to the first tested QTDT approach (Table 2, column F). We found that 135 genes were associated by using the VEGAS approach, but no causal genes were detected.

When we applied a linear model of association to the rare variants panel, we detected 68 genes pinpointed by 75 associated rare variants. This approach detected only two causal genes (*PIK3R3* and *PIK3C2B*) (Table 3, column G), and the increased sensitivity was accompanied by a reduction in the positive predictive value (Table 2, column G). When gene-based scores were established, 174 genes were considered associated, but no causal genes were detected. The variance component approach detected 71 genes, and the same 2 causal genes were identified (*PIK3R3* and *PIK3C2B*) (Table 3, column H). This approach presents a similar level of sensitivity and specificity as the other QTDT approaches (Table 2, column H). Using gene-based scores, we pinpointed a group of 27 genes but did not detect any causal genes. The limitation of the gene-based scores is directly related to the limited information regarding human rare variants in the current HapMap database.

## Discussion

Despite the relative success of GWAS in diseases such as and diabetes mellitus type 2 [11], their widespread use is rather limited in complex diseases. Markers identified by these studies explain only a small proportion of trait heritability.. Next-generation sequencing allows rare genetic variants (MAF < 0.01) of medical interest to be identified in any individual and those can be used to resolve the missing heritability paradox [12]. Because rare variants are almost family specific, their discovery and interpretation are best suited for family-based approaches [13]. There are many uncertainties about how to deal with rare variants; most approaches have been tested in case-control samples [2,14]. Collapsing methods summarize rare variant information using a counting method or a synthetic marker to capture the information provided by the haplotypic block underlying these variants [14]. In this report, we tested several statistical approaches to study rare variants using the familiar structure.

We serially tested and compared different ways to deal with the information provided by rare and common variants. We did not apply a multiple hypotheses testing correction in analyses because of the limited number of individuals in the cohort and consequent low power of the analysis. The threshold (0.05) is not realistic for deep-sequencing projects or GWAS where multiple-test correction is mandatory. Table 3 highlights the limitations of existing methods for dealing with rare genetic variants; these limitations are partly due to the use of a less informative binary trait. All tested approaches showed low sensitivity and low positive predictive value. The polygenic model using the information provided by common variants that alters trait heritability in at least one family presents the highest level of sensitivity but the lowest positive predictive value. It is noteworthy that some causal genes were detected by more than one approach. On the other hand, several causal genes (*AKT3*, *BCL2L11*, *NRAS*, *PIK3C3*, *PRKCB1*, and *RRAS*) were not detected using any of the explored methods (Table 3).

Biological information is progressively being added to regression models, allowing researchers to capture meaningful genetic information [15]. This premise was tested by the sole use of nonsynonymous variants information but was not successful endeavor (Table 2, columns A and B).. Although the use of pathway annotation databases has solid biological premises, it is seriously compromised by our limited knowledge. Only a third of the genes had entries in the KEGG database, and, consequently, any kind of annotation would have neglected a significant proportion of information. Gene-based association tests that use population-specific haplotypic substructure will probably be less affected by an isolated false-positive signal. This was not effective in our analysis because most of the rare variants present in the GAW17 data set are not cataloged in the HapMap database. With the completion of the 1000 Genomes Project, an impressive amount of rare variants will be detected and cataloged, making the approach more realistic. Information from rare variants is promising but requires a new generation of databases and tools to effectively to be mined in the next generation of genetic epidemiological projects.

## Conclusions

GWAS are the leading tool for identification of genetic markers that underlie phenotypes of interest. This information should be combined with rare variants identified by deep-sequencing projects. In this study, we serially tested different ways to deal with the information provided by rare genetic variants, and none was found to be especially superior to another. Our results could be seriously jeopardized by how the data were simulated or the limitation of the SOLAR package to deal with binary traits. But, based on our results, it is possible to pinpoint the necessity of new and more customized methods to deal with rare variants, especially in a family study design. In the next few years, we will be flooded with the data generated by deep-sequencing platforms, and these new methods will play a central role.

## Competing interests

The authors declare that there are no competing interests.

## Authors’ contributions

MAAA carried out computational, statistical analysis and drafted the manuscript. ARVRH carried out statistical analysis. PSLO participated in experimental design of the study; JEK: participated in experimental design and the development of the study; ACP: conceived the study and helped to draft the manuscript. All authors read and approved the final manuscript.
